# P-1972. Post-Marketing Surveillance of Hypersensitivity Reactions to Beta-Lactam Antibiotics Using the FDA AERS Database

**DOI:** 10.1093/ofid/ofaf695.2139

**Published:** 2026-01-11

**Authors:** Linta Susan Kuriakose, Albin C Sebastian, Alvin Sunny

**Affiliations:** Square Hospital, West Panthapath, Dhaka, Bangladesh; Square Hospital, West Panthapath, Dhaka, Bangladesh; Square Hospital, West Panthapath, Dhaka, Bangladesh

## Abstract

**Background:**

Beta-lactam antibiotics are among the most commonly prescribed antimicrobials worldwide, but they are also a frequent cause of drug-induced hypersensitivity reactions, ranging from mild rashes to life-threatening anaphylaxis. While clinical trial data provide initial safety signals, rare and delayed adverse events often emerge post-marketing. This study aimed to analyze post-marketing reports of hypersensitivity reactions associated with beta-lactam antibiotics using the U.S. FDA Adverse Event Reporting System (FAERS) database.

**Figure:**
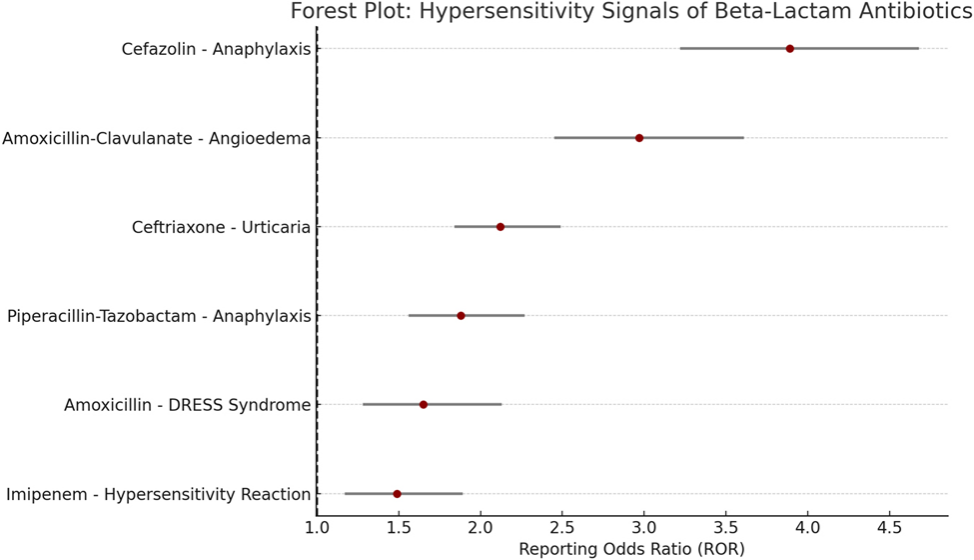
Forest Plot: Hypersensitivity Signals of Beta-Lactam Antibiotics (FAERS, 2010–2023)

This forest plot displays the reporting odds ratios (RORs) with 95% confidence intervals for hypersensitivity-related adverse events associated with beta-lactam antibiotics. Notable signals include:

Cefazolin–associated anaphylaxis (ROR 3.89)

Angioedema with amoxicillin-clavulanate (ROR 2.97)

Urticaria with ceftriaxone (ROR 2.12)

All events exceeding an ROR of 1 with lower CI >1 are considered statistically significant safety signals, reinforcing the need for careful antibiotic selection, particularly in sensitized patients.

**Methods:**

A retrospective pharmacovigilance analysis was conducted using FAERS data from January 2010 to December 2023. Reports involving penicillins, cephalosporins, carbapenems, and monobactams were extracted using generic and brand names. Hypersensitivity-related Preferred Terms (PTs) were identified via the MedDRA hierarchy, including anaphylactic reaction, angioedema, urticaria, erythema multiforme, and DRESS syndrome. Disproportionality was assessed using Reporting Odds Ratio (ROR) and Proportional Reporting Ratio (PRR), with statistical significance defined as ROR 95% CI >1 and ≥3 cases.

**Results:**

A total of 14,728 hypersensitivity-related adverse event reports were associated with beta-lactam antibiotics. Among them, amoxicillin accounted for the highest number of reports (n = 4,615), followed by ceftriaxone (n = 2,763) and piperacillin-tazobactam (n = 1,980). Disproportionality analysis showed the strongest signal for anaphylaxis with cefazolin (ROR: 3.89, 95% CI: 3.22–4.68) and angioedema with amoxicillin-clavulanate (ROR: 2.97, 95% CI: 2.45–3.61). Cephalosporins were more frequently associated with skin-related hypersensitivity (e.g., urticaria, erythema), whereas carbapenems were linked to a lower but consistent signal for severe reactions.

**Conclusion:**

This large-scale post-marketing analysis using FAERS data highlights distinct hypersensitivity risk profiles among beta-lactam subclasses. The findings reinforce the need for careful history-taking and individualized antibiotic selection, especially in patients with prior allergy history. Real-world data from spontaneous reporting systems like FAERS remain crucial in identifying emerging safety signals.

**Disclosures:**

All Authors: No reported disclosures

